# Uniting the nations of science

**DOI:** 10.7554/eLife.44441

**Published:** 2018-12-20

**Authors:** Eve Marder

**Affiliations:** 1 Volen CenterBrandeis UniversityWalthamUnited States; 2 Biology DepartmentBrandeis UniversityWalthamUnited States

**Keywords:** Living Science, teaching, diversity, creativity in science

## Abstract

As the world becomes smaller and more uniform, it is important to remember that creativity in science can happen anywhere.

Those of you who have seen any of the documentaries in which Anthony Bourdain traveled around the world eating, filming and talking with people of all nationalities, have undoubtedly been struck by many of the universalities of the human experience. At the same time, if you watch any of these films (and you should, if you have not), you will also be struck by the images and sounds of each location. Even in 2018, despite the ubiquitous reach of iPhones and Coca Cola, the streets and vistas of Provincetown on Cape Cod are very different from those of Kenya, Nepal and Lisbon. This remains, despite the proliferation of certain brands across so much of the world.

While science itself is universal, there are vast cultural and historical traditions, that even in this time of Skype and email, influence how science and science education are done around the world. Even substantial differences between Canadian, British, Australian and American scientific and educational cultures remain. These differences pale when compared to those countries whose first spoken language is not English, and whose first educational experiences are substantively different from those practiced in the US or UK. These cultural and historical differences continue to contribute to how we approach some of the most fundamental problems in biology. Of course, the internet makes it possible for students across the world to see me lecture, or to teach themselves linear algebra. And many of us travel around the world, but does a three-day tourist to Rome understand the depths and paradoxes of Italian culture? Does my week-long visit to labs and universities in India or China or Chile give me more than a very superficial sense of the scientific culture there? Indeed, I spent three years as a postdoc in Paris many years ago, and it took me longer to understand the differences in career expectations and ways of approaching a scientific problem in Paris than it took me to learn appropriate swear words in French.

Many years ago a Boston politician, Tip O’Neill, said: "All politics are local". Of course in 2018 that is both true and not true. The same can be said for science. Each of us does science and/or is part of an educational system in a local environment. Even such basics as what is needed in a curriculum to train a biologist is vastly different across institutions, states or provinces, and countries. Certainly the approaches to elementary and secondary education across the world appear to be quite different, as are societal attitudes to children who struggle to learn to read or do arithmetic. These differences are perhaps even more accentuated as we look at university education, with vast contrasts between the highly personal education offered at elite institutions in some countries and the public universities with large enrollments that can be found elsewhere.

Americans are sadly aware that many countries, much smaller than ours, are substantially better at teaching math and physics than we are. Some locales are specialists in educating computer scientists, and others plant biologists. There are many universities where there is no one who still knows how to identify butterflies, but hopefully there are other institutions where knowledge of mosses, ferns and spiders persists. Some of this speciation in knowledge is driven by the environmental circumstances, with some institutions under pressure to solve important technological challenges or to create a workforce with defined characteristics. There are countries that must deal with the control of infectious pathogens of crops or humans, and may not feel they have the luxury to develop basic science that does not directly contribute to solving agricultural or health concerns.

**Figure 1:**
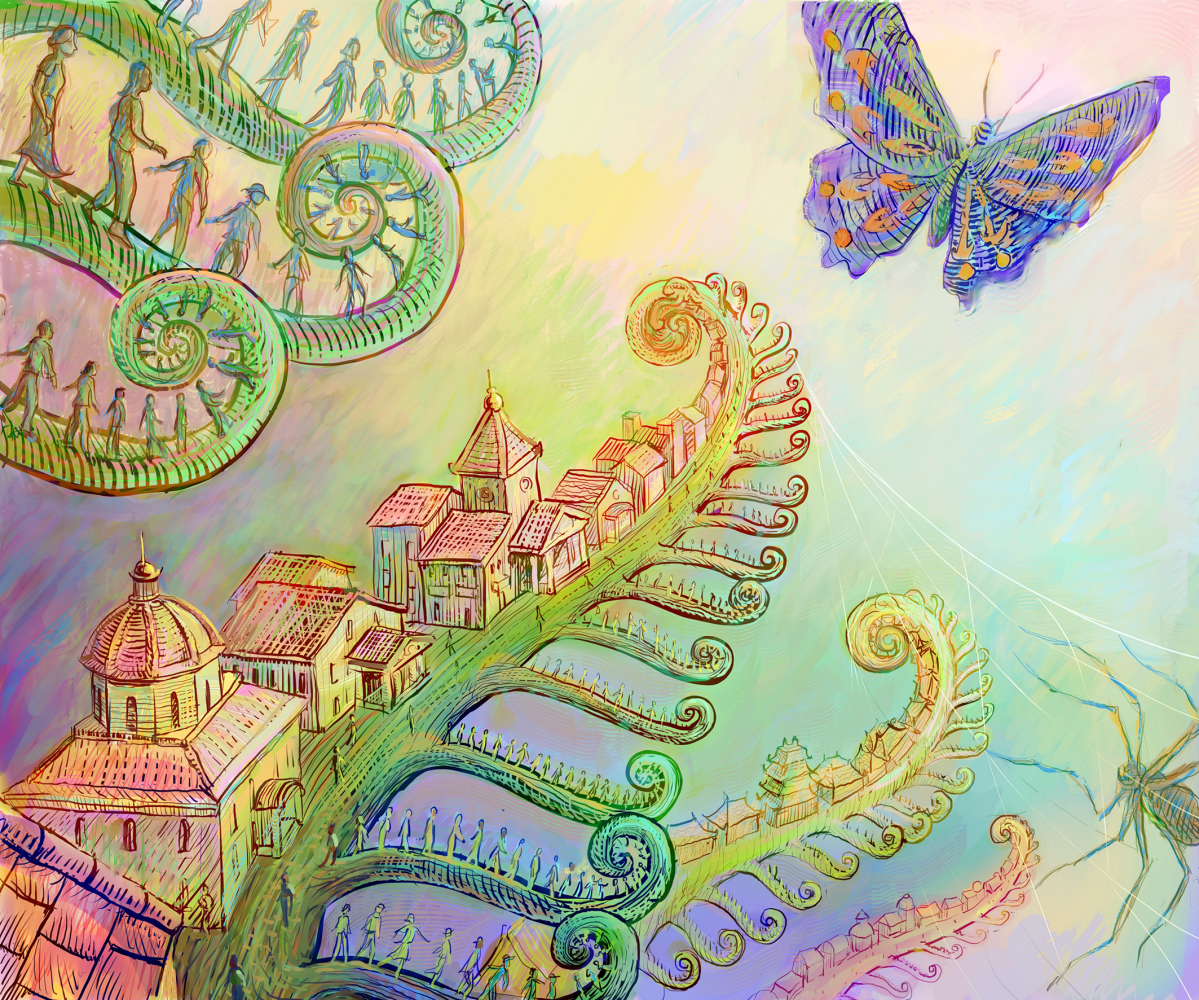
Just as biologists are taught to revel in the diversity of species that resulted from evolution, they should also appreciate the benefits that emerge from different approaches to education and research.

Illustration: Ben Marder.

As I was starting in science, there were strong traditions of excellence in some fields, so that the best of insect neuroethology might have been found in Germany, but the best of plant biology elsewhere. Certainly, as excellence attracts the best students, it is understandable that this kind of scientific speciation led to centers of excellence that were differentially distributed across the world. And even today, surprisingly, there are traces of the local scientific traditions of the past 150 years that persist in how science is done.

When I first traveled in Europe at the age of 20, it was possible to tell the nationality of people my age by their shoes, clothes and haircuts. Today, it is almost impossible to do so. The upside of open-access journals, bioRxiv and electronic communication is that information can be transferred very quickly and, like Starbucks, can infiltrate almost anywhere. The downside of all this is that it creates a strong impetus towards consensus science, or a push for scientists across the globe to consider the same problems interesting, and to employ the same approaches and methodologies in their research. When communication among scientists across the globe was slow and intermittent, small, semi-isolated communities were able to establish their own norms and opinions of what problems were interesting, and what methods considered necessary and sufficient.

In today’s world of lightning fast communication, many fields have developed recipes of what a paper must contain to be considered of sufficient quality for publication in an excellent journal. Some of the ingredients make sense (all papers should have appropriate statistics and adequate sample numbers), but others are now followed slavishly by authors, reviewers and editors without regard to whether they are appropriate for the problem at hand. My fear is that the unintended consequence of the drive for standards of excellence can result in loss of appreciation of papers and work that offer new insights without necessarily meeting all of the expected components of a paper in a specific field. At the end of the day, work from creative scientists who are formulating new approaches to scientific problems should be evaluated in terms of what new insights they have brought with their work, but not merely by whether their work has all of the components or attributes that the field expects.

Just as it pains me to see Coca Cola wherever I travel, it would be extremely sad if increased communication among biologists led to the loss of the study of idiosyncratic problems or species found only in some locales. Biologists are taught to revel in the diversity of species that resulted from evolution. Likewise, we should remember to treasure the opportunities to understand fundamental biological principles that result from those species and the diversity that remains in our training. Many of you contribute money and time to preserving endangered species in the world. Perhaps more of us should reflect on what we lose if we allow the loss of diversity in our scientific culture!

Anthony Bourdain made the universal shine through whether he was dining in a fine restaurant or eating street food in a crowded Asian market. Likewise, we should be able to appreciate and understand the lessons that come from experiments and measurements of clever and creative people, whether or not they follow the recipes established by consensus science.

